# Differential Impact of the HEN1 Homolog HENN-1 on 21U and 26G RNAs in the Germline of *Caenorhabditis elegans*

**DOI:** 10.1371/journal.pgen.1002702

**Published:** 2012-07-19

**Authors:** Leonie M. Kamminga, Josien C. van Wolfswinkel, Maartje J. Luteijn, Lucas J. T. Kaaij, Marloes P. Bagijn, Alexandra Sapetschnig, Eric A. Miska, Eugene Berezikov, René F. Ketting

**Affiliations:** 1 Hubrecht Institute–KNAW and University Medical Centre Utrecht, Utrecht, The Netherlands; 2 Wellcome Trust/Cancer Research UK Gurdon Institute, University of Cambridge, Cambridge, United Kingdom; Stanford University Medical Center, United States of America

## Abstract

RNA interference (RNAi)–related pathways affect gene activity by sequence-specific recruitment of Ago proteins to mRNA target molecules. The sequence specificity of this process stems from small RNA (sRNA) co-factors bound by the Ago protein. Stability of sRNA molecules in some pathways is in part regulated by Hen1-mediated methylation of their 3′ ends. Here we describe the effects of the *Caenorhabditis elegans* HEN1 RNA–methyl-transferase homolog, HENN-1, on the different RNAi pathways in this nematode. We reveal differential effects of HENN-1 on the two pathways that are known to employ methylated sRNA molecules: the 26G and 21U pathways. Surprisingly, in the germline, stability of 21U RNAs, the *C. elegans* piRNAs, is only mildly affected by loss of methylation; and introduction of artificial 21U target RNA does not further destabilize non-methylated 21U RNAs. In contrast, most 26G RNAs display reduced stability and respond to loss of HENN-1 by displaying increased 3′-uridylation frequencies. Within the 26G RNA class, we find that specifically ERGO-1–bound 26G RNAs are modified by HENN-1, while ALG-3/ALG-4–bound 26G RNAs are not. Global gene expression analysis of *henn-1* mutants reveals mild effects, including down-regulation of many germline-expressed genes. Our data suggest that, apart from direct effects of reduced 26G RNA levels of *henn-1* on gene expression, most effects on global gene expression are indirect. These studies further refine our understanding of endogenous RNAi in *C. elegans* and the roles for Hen1 like enzymes in these pathways.

## Author Summary

Small RNAs (sRNAs) have been shown to be potent regulators of gene expression in many different systems. They act by providing sequence specificity to Argonaute (Ago) proteins that in turn affect the expression and/or stability of mRNAs, or affect chromatin structures through recognition of nascent transcripts. Stability of sRNAs can be regulated by methylation of their 3′ end. This modification prevents addition of uridine residues that can destabilize the sRNA. The enzyme that catalyzes the methylation of sRNAs has been identified in Arabidopsis: HEN1. We describe studies on the *C. elegans* homolog of Hen1, *henn-1*. Our findings show that HENN-1 protein does not stably associate with the Ago proteins binding methylated sRNAs, but that HENN-1 does localize to subcellular regions known to host these factors. We find that the two known methylated sRNA species in *C. elegans* (21U and 26G) respond differently to loss of *henn-1*. While HENN-1 is required for 26G RNA stability in the germline, it has limited impact on 21U RNAs. In addition, we demonstrate that only ERGO-1–bound 26G RNAs are methylated, while those bound by ALG-3/4, are not. Our findings further refine the general understanding of 21U and 26G RNA pathways and identify two separable effects of HENN-1 on these RNAi–related mechanisms.

## Introduction

RNA silencing pathways are sequence-specific mechanisms acting in gene regulation and genome protection [1]–. Characteristic properties of these pathways are the involvement of a protein of the Argonaute (Ago) family that derives its sequence specificity from a bound small RNA (sRNA) molecule [6], [7]. Many different Ago paralogs exist and the type of Ago protein acting in a particular pathway will dictate the effect of that RNA silencing pathway. Ago proteins have a characteristic domain structure. They consist of a rather variable N-terminal region, followed by PAZ, MID and PIWI domains. The PAZ and MID domains function in binding the 3′ and 5′ ends of the sRNA co-factor respectively. The sRNA runs through the PIWI domain where it is exposed such that it can readily engage in base-pairing with potential target RNA molecules. The PIWI domain itself has an RNaseH-like fold and can be involved in direct endo-nucleolytic cleavage of the targeted RNA. However, in some Ago paralogs amino-acids at key catalytic positions are incompatible with hydrolysis, most likely crippling enzymatic activity.

sRNA molecules acting in RNA silencing can be formed through several biochemical routes involving either double stranded (ds) or single stranded (ss) RNA precursors [5], [8]. When dsRNA is involved, the enzyme Dicer usually produces sRNAs, a reaction that has been well characterized. Pathways using ssRNA as a substrate for the production of sRNAs are much less understood. In some pathways endo-nucleases create the 5′ ends of new sRNAs. Presumably, this 5′ end is then bound by an Ago protein after which the 3′ end of the RNA is trimmed to fit the Ago protein [1]. In other pathways, RNA dependent RNA polymerases (RdRP) synthesize stretches of RNA that may be directly bound by Ago proteins [9]–[11]. This type of sRNA is characterized by a 5′-tri-phosphate group, likely derived from the first nucleotide used by the RdRP enzyme.

Some types of sRNA are methylated on the 2′hydroxyl group of their 3′ terminal nucleotide. The enzyme responsible for this, HEN1, has been first described in plants [12]. Following this discovery, HEN1 homologues have been described in *Drosophila* [13], [14], mouse [15], *Tetrahymena* [16] and zebrafish [17]. Biochemically, HEN1 activity has been intimately linked with piRNA biogenesis; it likely acts on the piRNA precursor while it is already bound by a Piwi protein [18]. Consistent with this idea, miR-277 in *Drosophila* can be found methylated or not depending on whether it has been loaded into Ago1 or Ago2 [19]. Ago proteins hosting methylated sRNAs have an adapted PAZ domain that allows and prefers a 2′-O-methylated base at the 3′ end of the sRNA [20]. Functionally, it has become clear that 3′ end methylation can protect sRNAs from the non-templated addition of uridine residues [17], [19] that in turn have been suggested to trigger degradation of sRNAs [12], [21], [22]. Uridylation of sRNAs has been shown to be especially relevant for sRNAs displaying extensive complementarity with their targets, reaching out to their 3′ ends [19]. The mechanism behind this observation likely involves release of the 3′ends of such highly complementary sRNAs from the PAZ domain, making them accessible for 3′end modifying activities.

In the nematode *C. elegans* a large number of endogenous RNA silencing (endoRNAi) pathways has been identified, each being characterized by specific types of Ago proteins and their bound sRNA cofactors. One of these is the highly conserved miRNA pathway, mediated by the Ago proteins ALG-1 and ALG-2. Other RNA silencing pathways in *C. elegans* are the 26G, 21U and 22G pathways. These names reflect the size of the sRNA co-factors involved and the prevalence of G or U residues at their 5′ ends. In oocytes and embryos, the Piwi-related Ago protein ERGO-1 binds 26G RNAs [23], [24], while during spermatogenesis the ALG-1/2-related Ago proteins ALG-3 and ALG-4 act as 26G RNA hosts [24], [25]. The *C. elegans* Dicer homolog DCR-1 has been shown to be required for 26G RNA production, presumably using dsRNA that is produced through the action of the RNA dependent RNA polymerase (RdRP) enzyme RRF-3 [26], [27]. The targets of the 26G pathways are readily identified as they display perfect sequence complementarity to the genes from which they are derived.

21U RNAs [28] are bound by the Piwi-type Ago proteins PRG-1 and PRG-2 [29], [30]. The biogenesis of 21U RNAs is poorly understood. Their genomic templates are clustered in specific regions on one chromosome and each 21U locus is characterized by a specific upstream motif that may serve either as an RNA processing signal or as a promoter element [28]. No further components related to 21U RNA biogenesis have been identified. For most 21U RNAs no perfectly complementary sequences other than their regions of origin are present in the *C. elegans* genome. A small number of 21U RNAs target the DNA transposon Tc3, resulting in 22G RNA production from Tc3 elements [30]. However, in general terms, it is difficult to predict targets of 21U RNAs purely based on sequence homology. Phenotypes of *prg* mutants [29]–[31], however, indicate that these Piwi proteins do have endogenous functions and that therefore 21U RNAs do have endogenous targets. Indeed, in recent work, Bagijn *et al.* identify 21U RNA targets displaying varying degrees of target complementarity [32].

Both 26G and 21U pathways can be considered as primary RNAi pathways, as they have been shown to trigger the production of secondary 22G RNAs by activating the RdRP enzymes RRF-1 and EGO-1 on targeted mRNAs [23]–[25], [29], [30], [32], [33]. 22G RNA production can also be initiated through other triggers, such as exogenous RNAi, i.e. RNAi triggered through exogenously introduced dsRNA. In this case RDE-1 acts as the primary Ago protein, after which the secondary 22G RNAs are fed into a variety of secondary Ago proteins [34], [35]. In other cases, such as in the CSR-1 mediated 22G pathway [36], a primary Ago protein has not yet been identified. Nonetheless, EGO-1 and RRF-1 are still required for the production of CSR-1-bound 22G RNAs. Targets of 22G pathways are readily identified, as 22G RNAs display perfect complementarity to the genes from which they originate. Phenotypes of mutants in these various pathways are diverse. In some cases, multiple Ago proteins have to be mutated in order to visualize phenotypic defects [34], in other cases mutation of a single Ago protein already induces an effect [35]–[37].

The two endogenous primary sRNA classes in *C. elegans* that have been described to date, 26G and 21U RNAs, are 2′O-methylated [28]. In order to reveal the impact of this 3′-end modification is on these two sRNA types, we here describe studies on the *C. elegans* HEN1 homologue, HENN-1.

## Results

### HENN-1 is the *C. elegans* homolog of Hen1

**Figure 1:**
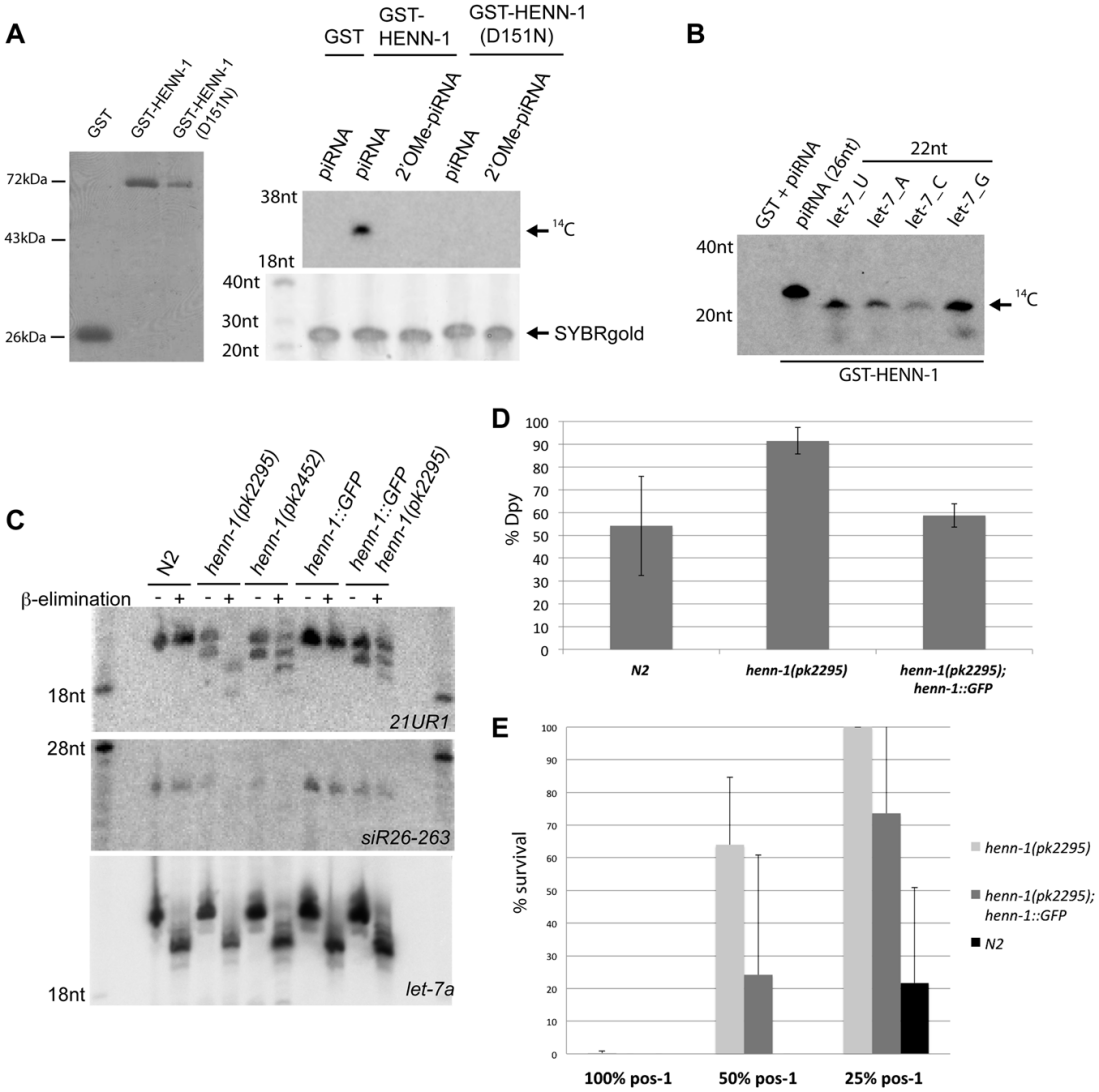
HENN-1 is the *C. elegans* homolog of Hen1. (A) Left panel: protein gel stained with PageBlue shows purified GST, GST-HENN-1 and GST-HENN-1(D151N) proteins used to perform methyltransferase assays as shown in the right panel and B. Right panel: *in vitro* methyltransferase activity assay. RNA oligos were incubated with indicated proteins and 14C-labelled SAM. Reaction products were run on a 12% acryl-amide gel. (B) *In vitro* methyltransferase assay using different RNA substrates each differing in the identity of the most 3′ nucleotide. (C) Northern blot analysis using RNA from wild-type, *henn-1(pk2452)*, *henn-1(pk2295)* and *henn-1(pk2295); pgl-3:HENN-1::GFP* animals. Blots were probed for 21UR1 and 26G species siR26-263. Probing for *let-7* serves as loading and as oxidation-β-elimination control. (D) Response of wild type (N2), *henn-1(pk2295)* and *henn-1(pk2295); pgl-3:henn-1:GFP* to *dpy-13* RNAi. *Henn-1(pk2295)* sensitivity is significantly higher than both controls (two-tailed t-test, n = 5: p<0.05 for both). (E) Response of wild type (N2), *henn-1(pk2295)* and *henn-1(pk2295); pgl-3:HENN-1::GFP* to *pos-1* RNAi, delivered at three different dosages: undiluted (100%), diluted one to one (50%) and diluted one to four (25%). At 50% *pos-1* RNAi, *henn-1(pk2295); pgl-3:HENN-1::GFP* animals display significant rescue (p = 0.01) of the *henn-1(pk2295)* RNAi defect (p<0.0005). The p-values at 25% *pos-1* RNAi are p = 0.04 for both the *henn-1(pk2295)* RNAi defect and the rescue. P-values were calculated with a two-tailed t-test, n = 10.

Based on homology, C02F5.6 is the *C. elegans* homolog of *Arabidopsis* HEN1 [38] (Figure S1). We purified a recombinant GST-tagged version of C02F5.6 and tested this protein for methylating activity on the 3′ end of ssRNA molecules (Figure 1A). Indeed, C02F5.6 can methylate sRNA molecules *in vitro* and this activity is blocked by the presence of a 2′O-methyl group at the terminal 3′ base of the substrate (Figure 1A). This reaction does not display major sensitivity to the identity of the terminal 3′ base of the substrate (Figure 1B). We named C02F5.6 *henn-1*, for HEN1 of nematode-1.

To study HENN-1 *in vivo*, we isolated two *C. elegans* strains carrying mutant alleles of C02F5.6 [39], and out-crossed them with wild-type animals. Both *henn-1* alleles result in slightly reduced brood size counts and normal survival (Figure S2). One allele, *pk2295*, introduces a premature stop at position 347, in the C-terminal part of the protein. The other allele, *pk2452*, introduces a missense mutation in the catalytic domain of HENN-1, changing the aspartic acid at position 151 to an asparagine (Figure S1). This effectively replaces a carboxyl group (COOH) for an amide group (CONH2). Through structural analysis [40], the homologous residue in *Arabidopsis* HEN1 (D745) has been shown to be involved in binding the AdoMet/AdoHcy cofactor and therefore *pk2452* represents a good candidate allele for inducing loss of HENN-1 activity. We tested this by making recombinant GST-HENN-1(D151N), and subjected this protein to our *in vitro* RNA methylation assay (Figure 1A). This revealed reduced activity of HENN-1(D151N), suggesting that cofactor binding or release may indeed be affected.

To probe the effects of these two *henn-1* alleles *in vivo* we analyzed 21U RNAs using Northern blotting, probing for 21U species 21UR1 (Figure 1C). In both *henn-1* alleles a faster migrating band appears below the mature 21UR1 form, suggesting that a fraction of the 21U RNAs become shorter when HENN-1 activity is compromised. We also checked for the presence of 2′O-methylation on 21UR1 by applying oxidation followed by mild alkaline treatment. This procedure removes the ultimate 3′ base from non-modified RNA molecules, leaving a 3′ phosphate, while modified sRNAs such as 21U RNAs are resistant [41]. Indeed, wild-type 21UR1 is resistant to this treatment but 21UR1 isolated from *henn-1(pk2295)* animals is fully sensitive (Figure 1C). *Henn-1(pk2452)*-derived 21U RNA displays partial sensitivity to this procedure, indicating that *henn-1(pk2452)* is a hypomorphic allele of *henn-1*. These defects are partially rescued by transgenic GFP::HENN-1 expression driven by a heterologous promoter (*pgl-3*) (Figure 1C).

We also tested whether 26G RNAs are affected by HENN-1 (Figure 1C). Northern blotting revealed that the stability of 26G-263 is significantly affected, preventing robust detection of 26G-263 after oxidation and β-elimination. However, overexposure of the blot does reveal a very weak signal at the height where we would expect unprotected 26G-263 to run after this treatment (Figure S3). Taken together we conclude that HENN-1 can methylate RNA *in vitro* and is required for 21U and 26G RNA methylation *in vivo*.

### Exogenous RNAi is affected in *henn-1* mutants

Since HENN-1 methylates 26G RNAs, and the 26G RNA pathways have been found to interact with exogenous RNAi (exoRNAi) [23], [24], [42]–[45], we asked whether *henn-1* mutants displayed exoRNAi defects. RNAi against *dpy-13* (somatic RNAi) revealed an enhanced RNAi phenotype (Eri) in the soma of *henn-1(pk2295)* mutants (Figure 1D). This can be rescued by expressing HENN-1::GFP from a *pgl-3* promoter. Similar Eri phenotypes can also be observed upon RNAi against *sqt-3*, *lir-1* and *pop-1* (Figure S4). In contrast, RNAi against the germline gene *pos-1* reveals a mild, but significant RNAi defective (Rde) phenotype that is partly rescued by *pgl-3* driven HENN-1::GFP (Figure 1E). The Rde phenotype is not specific for *pos-1*, as we observe comparable results when we perform RNAi against another germline expressed gene, *gpb-1* (Figure S4). This is, to our knowledge, the first *C. elegans* mutant that displays a mixed Eri/Rde phenotype.

### HENN-1 expression analysis

**Figure 2:**
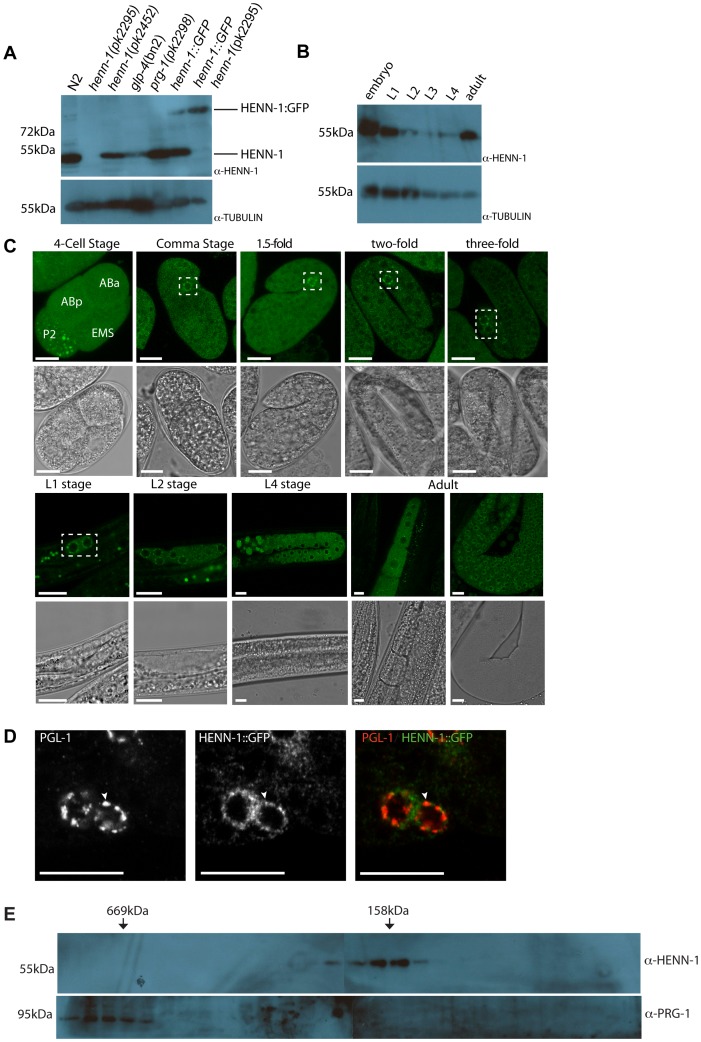
HENN-1 expression analysis. (A) Western blot analysis for HENN-1 expression in different mutant backgrounds. *Glp-4(bn2)* animals contain almost no germ cells. Tubulin is shown as loading control. (B) Western blot analysis for HENN-1 expression at different time points during development of *C. elegans*. *Glp-4(bn2)* animals contain almost no germ cells. Tubulin is shown as loading control. (C) Confocal images (single z-plane) of HENN-1::GFP expressing animals at different developmental stages. Nuclei of embryonic germ cells are outlined in white boxes. Blastomere identities in the four-cell stage embryos are indicated. Scale bars are 10 µm. (D) Immuno-fluorescence with anti-PGL-1 and anti-GFP antibodies. The white arrowhead indicates a site of co-localization. Scale bars are 10 µm. (E) Western blots for HENN-1 (top) and PRG-1 (bottom) on fractions obtained after gel filtration.

To analyze endogenous HENN-1 protein we raised antibodies to a peptide representing the very N-terminal part of HENN-1. On Western blots, affinity purified fractions of this antibody recognize a protein of roughly the correct size in protein extracts from wild-type animals, while *henn-1(pk2295)* mutant extracts lack this protein (Figure 2A). Interestingly, *henn-1(pk2452)* animals produce normal amounts of HENN-1 (Figure 2A), suggesting that the loss of 21U RNA methylation described above indeed results from reduced enzyme activity, rather than from reduced HENN-1 stability. On Western blots we can detect HENN-1 in all stages of wild-type animals, with strong expression in embryos and adults (Figure 2A, 2B). Also animals that lack germ cells (*glp-4* mutants) express HENN-1, albeit at a reduced level (Figure 2A). These data indicate that HENN-1 is expressed rather ubiquitously, with strong expression in the germline and embryos.

In order to analyze the subcellular localization of HENN-1, we expressed transgenic, GFP-tagged HENN-1 in the germline from a heterologous promoter (*pgl-3*) and analyzed expression using confocal microscopy (Figure 2C). As described above, this C-terminal GFP fusion is functional and is expressed at levels similar to those of endogenous HENN-1 (Figure 2A). In early embryos we find HENN-1::GFP rather diffuse in the cytoplasm, with the exception of the P-lineage, the progenitors of the future germ cells. In this lineage we find HENN-1::GFP in discrete granules (Figure 2C). At later stages, HENN-1::GFP localizes to peri-nuclear granules in the germ cells. This includes the Z2 and Z3 cells, the proliferating germ cells in L2–L3 animals and the germline nuclei in the adult gonad. These granular structures seem to disperse upon differentiation of germ cells into oocytes (Figure 2C). Interestingly, in zebrafish we also observe diffuse Hen1 localization in oocytes (not shown), which is followed by nuage-bound Hen1 during embryogenesis [17], suggesting that this may be an evolutionary conserved property of HEN1-like enzymes. It should, however, be noted that some non-HENN-1-associated GFP protein may be present as we can detect a potential GFP-only signal in Western blots of HENN-1::GFP transgenic animals (Figure S5).

P-granules are known to host many RNAi pathway components including PRG-1 [29]. We checked whether the embryonic HENN-1::GFP-positive granules co-localize with P-granules, using PGL-1 as a marker [46]. This revealed that PGL-1 and HENN-1::GFP often co-localize (Figure 2D), although many HENN-1::GFP and PGL-1 foci appear to be adjacent rather than fully overlapping.

### HENN-1 does not form a stable complex with PRG-1

To probe the molecular surroundings of HENN-1 we subjected embryonic extracts to size-exclusion chromatography. The resulting fractions were analyzed by Western blotting, probing for HENN-1. This revealed that HENN-1 is present in a complex of roughly 150 kDa (Figure 2E). Given the approximately 50 kDa size of HENN-1 itself these data suggest that HENN-1 either is present as a tri-mer in our extracts, or partners with one or more additional proteins. A good candidate for such a HENN-1 binding protein is PRG-1, since the PRG-1 bound 21U RNAs are methylated and it has been previously shown that *Drosophila* Hen1 interacts physically with Ago proteins [14]. However, PRG-1 elutes from size-exclusion columns at much higher molecular weight than HENN-1 (Figure 2E). Furthermore, PRG-1 localization to P-granules is not affected by loss of HENN-1, as visualized by a GFP::PRG-1 reporter (Figure S6A) and yeast-two-hybrid experiments failed to show a direct interaction between HENN-1 and PRG-1 (data not shown). Finally, PRG-1 is present at normal levels in HENN-1 mutant animals (Figure S6B). We therefore conclude that HENN-1 is not a stable interaction partner of PRG-1 and is not required for PRG-1 stability and localization.

### HENN-1 enhances 21U RNA–mediated silencing

**Figure 3:**
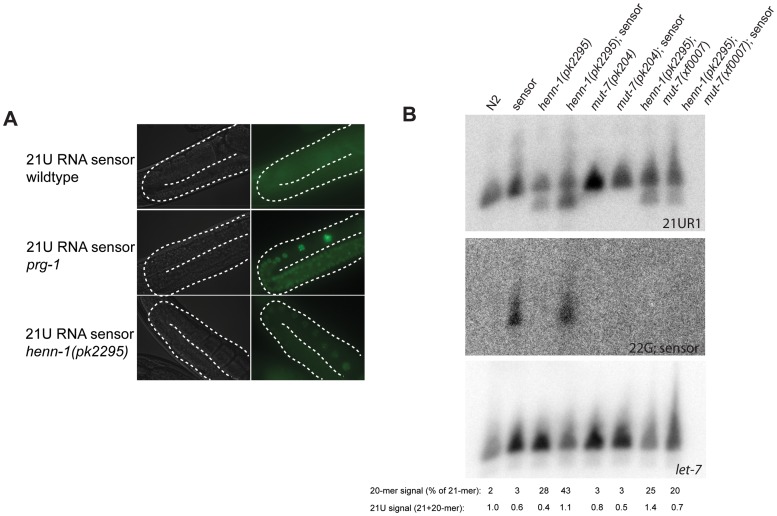
*henn-1* affects 21UR1 sensor activity. (A) Activity of a transgene expressing GFP (21UR1 sensor), silenced by 21U species 21UR1 in wild-type, *prg-1(pk2298)* and *henn-1(pk2295)* mutant backgrounds. The gonads are outlined with a white dashed line. (B) Northern blot analysis of 21UR1 in young adult animals of the indicated genotypes. “Sensor” refers to the 21UR1 sensor also shown in panel A. Signal intensities are related to *let-7*, and the 21UR1:*let-7* ratio in N2 is set at one. The 21-mer and 20-mer signals of the 21UR1 probe have also been quantified separately. The signal intensity of the 20-mer relative to the total 21UR1 signal is presented. Repetition of this blot with two independent biological samples has shown that the apparent differences are not reproducible. The 22G-sensor blot shows the signals obtained after hybridizing probes homologous to the 21UR1 sensor.

In *Tetrahymena*, flies and zebrafish, loss of Hen1 activity has been shown to impair Piwi pathway activity, accompanied by a reduction in piRNA levels [13], [14], [16], [17]. However, 21U RNA levels do not seem to be affected significantly in *henn-1* mutant animals. We therefore asked whether the silencing activity of the PRG-1 pathway is compromised in *henn-1* mutant animals. We first asked if the Tc3 transposon becomes activated in *henn-1(pk2295)* mutants, as Tc3 has been identified as a 21U RNA target [30]. We did this by testing the reversion frequency of a Tc3 insertion allele of the *unc-22* gene. In this assay we could not detect enhanced Tc3 activity in absence of HENN-1 (reversion frequency <10^−7^; data not shown). To probe the effects of *henn-1* on 21U targets further, we introduced a transgene that is silenced by 21UR1 through the presence of a 21UR1 complementary site in the 3′UTR of the mRNA encoding a GFP-Histone 2B fusion protein [32]. As expected, loss of PRG-1 de-silences this reporter (Figure 3A). Placement of the same transgene in *henn-1(pk2295)* (Figure 3A) or *henn-1(pk2452)* (data not shown) backgrounds results in a mild increase in GFP:H2B signal, indicating reduced silencing of the GFP reporter in absence of HENN-1 function.

### 21UR1 is not subject to target-dependent destabilization

As it has been shown that sRNA stability can be affected by the presence of target RNA molecules that display extensive complementarity [19], we checked 21UR1 levels in wild-type and *henn-1* mutant backgrounds in both absence and presence of the 21UR1 sensor. Using Northern blotting as a read-out we have been unable to find significant and consistent effects of 21UR1 target RNA on the levels of 21UR1 in wild-type and *henn-1* mutant animals. In Figure 3B a representative blot is shown. Also the relative amounts of 20-mer versus 21-mer species do not display consistent effects. An explanation for this observation could be that in *C. elegans* 21U mediated silencing proceeds via a downstream endoRNAi process [32]. This downstream step may reduce the levels of the 21UR1 sensor such that they become too low to trigger 21UR1 destabilization. We therefore repeated the experiment in a *mut-7* mutant background, in which the 22G RNA pathway downstream of PRG-1 is inactivated, resulting in strongly increased 21UR1 sensor activity [32]. Indeed, upon loss of *mut-7* the 22G RNAs derived from the sensor RNA disappear, but we still do not observe significant effects of the 21UR1 sensor on 21UR1 in *henn-1* mutant animals (Figure 3B). From these data we have to conclude that, in contrast to other sRNAs [19], 21UR1 stability is not strongly affected through target RNA recognition, even when the target RNA is perfectly complementary.

### HENN-1 has minor effects on global 21U RNA stability

**Figure 4:**
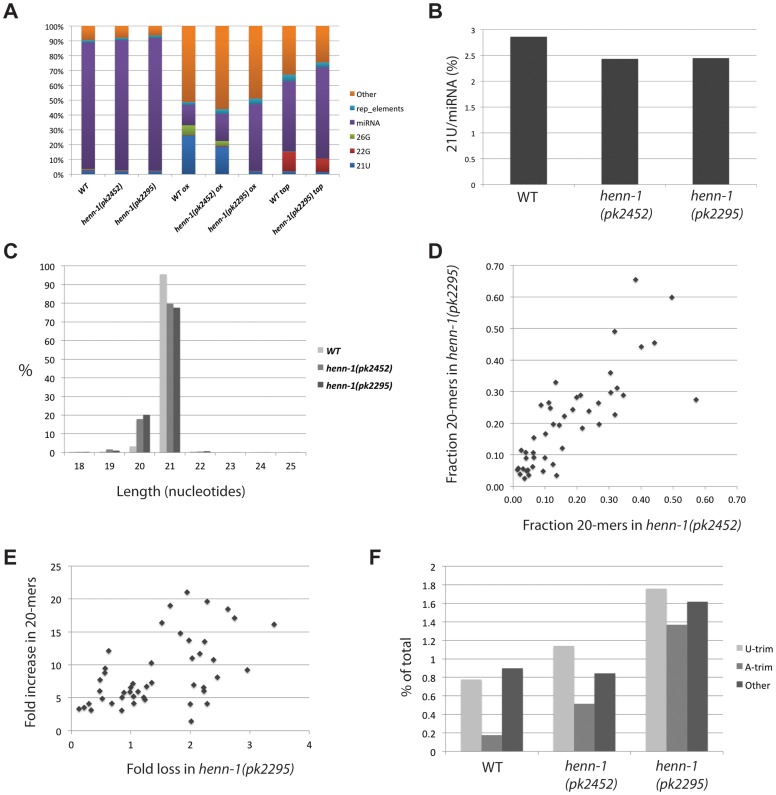
Global effects of *henn-1* on 21U RNAs. (A) Bar diagram displaying the different annotated small RNA reads obtained after deep-sequencing. Reads from structural RNAs were removed before analysis. (B) The expression level of 21U RNAs in wild-type and *henn-1* mutant backgrounds. Total 21U reads are normalized to total miRNA reads. The differences between wild-type and *henn-1* mutant samples are significant (Chi-squared test; p<10^−10^). (C) Length distribution plot of 21U RNAs. (D) Scatter plot displaying the fraction of 20-mer species of individual 21U RNA loci that were represented by at least 250 raw reads (20+21-mers) in each of the libraries used for this analysis (*henn-1(pk2452)* and *henn-1(pk2295)*). (E) Scatter plot displaying individual 21U loci represented by at least 250 raw reads (20+21-mers) in each of the libraries used for this analysis (wild-type and *henn-1(pk2295)*). X-axis: fold loss of reads in the *henn-1(pk2295)* background relative to wild-type. Y-axis: fold increase of 20-mer species in the *henn-1(pk2295)* background relative to wild-type. (F) Bar diagram displaying the frequencies of non-templated base additions found on 21U reads, as a percentage of the total 21U read count.

In order to study the effects of HENN-1 on sRNAs in general, we prepared sRNA libraries from wild type, *henn-1(pk2452)* and *henn-1(pk2295)* young adult animals. To enrich for 2′-O-methylated small RNAs we also made libraries of the same RNA preparations, but after oxidative treatment with NaIO_4_. After deep sequencing on an Illumina platform, we mapped the obtained reads to the *C. elegans* genome (WS220) and annotated miRNAs, 22G RNAs, 26G RNAs and other types of RNAs based on Ensemble database v.62 (Tables S1 and S2). We then expressed the read counts as reads per million (rpm), excluding reads matching to structural RNAs, such as rRNA and tRNA (Table S3). A bar diagram reflecting the abundance of the various identified sRNA classes in each of the libraries is depicted in Figure 4A.

As observed before [23], oxidation of the wild-type sample enriches for 21U RNAs. Consistent with the above described loss of 21U RNA methylation, this effect is less pronounced in the *henn-1(pk2452)* sample, and oxidation of RNA obtained from *henn-1(pk2295)* animals does not enrich for 21U RNA at all (Figure 4A). In the non-oxidized libraries we clone fewer 21U RNAs from *henn-1* mutant samples compared to wild-type (Figure 4B), but the overall loss of 21U RNAs is rather small. This is consistent with our Northern blotting data showing that 21UR1 levels do not decrease when HENN-1 is lost, but contrasts the fate of piRNAs in other systems where loss of HEN1 homologs has been described [12]–[14], [16], [17]. Again consistent with our Northern blotting data, we observe an increase of 20-nucleotide-long reads from 21U loci (Figure 4C). Since we only annotate a read as a 21U RNA when its 5′ end precisely matches annotated 21U RNAs, this indicates increased variability at the 3′ end upon loss of 2′-O-methylation. The frequency of 20-mers for individual 21U species varies significantly, ranging from as low as 3% to almost 70% (Figure 4D). Plotting the frequencies of 20-mer species observed in the *henn-1(pk2452)* background versus those observed in the *henn-1(pk2295)* background (Figure 4D) revealed a strong linear correlation (R^2^ = 0.7), with the 20-mer frequencies in the *henn-1(pk2295)* background slightly higher that that in the *henn-1(pk2452)* background. In addition, an overall weak, but positive correlation (R^2^ = 0.3) can be observed between the increase of 20-mer frequency of a given 21U species and the loss of that species upon disruption of *henn-1* (Figure 4E). From these results we draw the following conclusions. First, the frequency of 20-mer species of individual 21U RNA species is reproducible and likely reflects a specific phenomenon rather than intrinsic noise. Second, the appearance of 20-mer versions of a specific 21U RNA reflects the stability of that individual 21U RNA species. This is very similar to observations relating to piRNAs in absence of Hen1 in zebrafish [17], although the observed effects of *henn-1* on 21U RNAs are much weaker.

Next, we analyzed the identity of non-templated nucleotides at the 3′ end of 21U RNA reads that have been trimmed off during the mapping process. We classified these trimmed reads as either ‘U-trim’, in case only non-matching T-bases were found at the end of a sequence, ‘A-trim’ or ‘Other’. Normally, 21U RNAs are rather infrequently extended at their 3′ end, as reflected by the low percentage of trims (Figure 4F). In addition, there is no strong bias for U-trims. This contrasts to what we have described for zebrafish piRNAs [17]. Upon loss of HENN-1 activity 3′end extension frequencies increase, but still they do not display enrichment for U-trims (Figure 4F). Since uridylation has been proposed to correlate with destabilization, these findings are consistent with the observation that 21U RNA levels do not significantly decrease in *henn-1* mutant animals. Unfortunately, the low abundance of most individual 21U RNAs in our libraries, including those described to recognize Tc3 and 21UR1, prohibits meaningful analysis of uridylation frequencies in further detail.

### Differential effects of HENN-1 on ERGO-1– and ALG-3/4–bound 26G RNAs

Next, we analyzed 22G and 26G RNAs in wild-type and *henn-1* mutant samples. In order to increase the 22G fractions in our libraries we also prepared wild-type and *henn-1(pk2295)* libraries from TAP-treated RNA, a treatment that reduces 5′-triphosphate groups to mono-phosphate, allowing the ligation of 5′ adaptor oligos to the 22G class sRNAs. Both 22G and 26G RNAs are found as sub-populations of RNA molecules that we initially classify as ‘siRNA’: antisense reads mapping to annotated genes. To analyze 26G and 22G classes individually we extracted 22G and 26G RNAs from the siRNA group, asking for a length of 22 or 26 nucleotides respectively and the presence of a 5′G.

**Figure 5:**
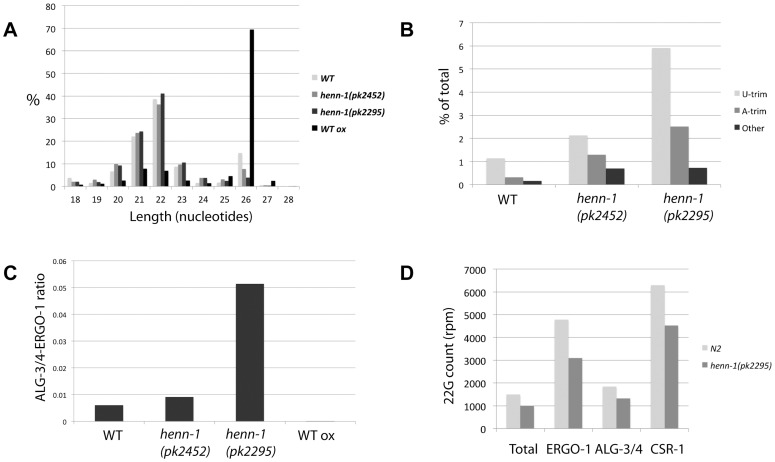
Effects of *henn-1* 22G and 26G RNAs. (A) Length distribution plots of ‘siRNA’ category, containing both 22G and 26G RNAs, in diverse libraries. (B) Bar diagram displaying the frequencies of non-templated base additions found on 26G reads, as a percentage of the total 26G read count. P<0.0001 for *henn-1(pk2452)* and *henn-1(pk2295)* relative to wild-type (Chi-squared test). (C) Ratio of ALG-3/4 and ERGO-1 bound 26G RNAs as derived by previously described annotation (see main text), in diverse libraries. P-values of all differences <0.001 (Chi-squared test). (D) The 22G counts, in rpm, for all genes in total, ERGO-1, ALG-3/4 and CSR-1 target genes. 22G count for ‘Total’ was divided by 100 for better visualization.

Plotting length distribution profiles of the siRNA class as a whole (Figure 5A) reveals that the 22 and 26 nucleotide sub-populations dominate in wild-type animals. Consistent with the finding that 26G RNAs are 2′-O-methylated, the 26 nucleotide peak increases when wild-type RNA is oxidized before cloning. Interestingly, 26 nucleotide reads are less prominent in *henn-1(pk2452)* mutants and almost completely drop to background levels in *henn-1(pk2295)* animals. This indicates that, unlike 21U RNAs, 26G RNAs strongly depend on HENN-1 for stability. This is also reflected in the frequency of uridylation of 26G RNAs in both wild-type and *henn-1* mutant animals (Figure 5B): in all samples U-trims are most abundant and U-trim frequencies rise sharply upon progressive inactivation of HENN-1. It should be noted, however, that also other types of non-templated extensions increase in frequency, including adenylation. These data show that 26G RNAs are less stable and more prone to non-templated nucleotide additions, including uridylation, when they are not methylated.

We further analyzed 26G RNAs with regard to their hosting Ago protein. To reduce potential background from 22G RNAs we only used the non-TAP treated libraries for these analyses. It has previously been shown that 26G RNAs can be divided into two classes: those binding to ERGO-1 [23],[24] and those binding to ALG-3 or ALG-4 (ALG-3/4) [24], [25]. We annotated our 26G reads as ERGO-1 or ALG-3/4 26G RNA (Table S4) based on these studies. We then asked whether both types of 26G RNA respond similarly to loss of HENN-1 by plotting the ratio of ALG-3/4 and ERGO-1 bound 26G RNAs. This revealed a striking increase in ALG-3/4 26G RNAs relative to ERGO-1 26G RNAs upon *henn-1* mutation (Figure 5C). While this could be caused by the fact that *henn-1* mutant strains are slightly Him, e.g. they have more males in their progeny than wild-type strains, we also observed a decrease of ALG-3/4 26G RNA cloning frequencies upon oxidative treatment of the RNA, indicating that they are not methylated (Figure 5C). Together, these data strongly suggest that 26G RNAs are only methylated in the context of ERGO-1, and not in the context of ALG-3/4.

Finally, we addressed the behavior of 22G RNAs based on the wild-type and *henn-1(pk2295)* TAP treated libraries. Global levels of 22G RNAs are roughly 30% lower in *henn-1(pk2295)* animals (Figure 5D and Figure S7), without indications for major changes in size distribution (Figure 5A) or uridylation frequencies (Figure S8). To check whether the global decrease of 22G RNAs is caused directly by lower ERGO-1 26G RNA levels we asked whether the 22G RNA counts of the ERGO-1 pathway, the ALG-3/4 pathway and the CSR-1 pathway (a 26G RNA unrelated endogenous RNAi pathway) are differentially affected by *henn-1* mutation. Since CSR-1 targets many genes, we selected the top-ranking CSR-1 targets, asking for a cloning frequency of at least 500 rpm and a cloning ratio of at least 0.9 from CSR-1 IPs [36] (Table S3). Surprisingly, the 22G RNA populations of all these gene categories go down roughly 30% (Figure 5D). Thus, while ERGO-1 mediated 22G RNA biogenesis may be directly affected by loss of HENN-1 and the subsequent loss of ERGO-1 bound 26G RNAs, our data does not provide direct support for this hypothesis.

### HENN-1 affects global germline expression levels

**Figure 6:**
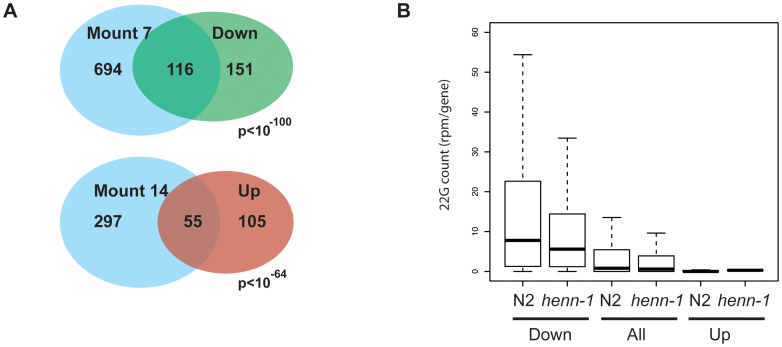
Gene expression analysis of *henn-1* mutants. (A) Venn diagram showing the overlap between up- and down-regulated genes and the gene expression domains, as defined by Kim *et al.* [47], for which they are most enriched. The Holm-Bonferroni corrected p-value is given. ‘Mount 7’: Germline–enriched. ‘Mount 14’: Collagen. (B) Box-plot displaying the 22G coverage per gene in wild-type and *henn-1(pk2295)* mutant libraries. Genes are grouped into ‘Down’ and ‘Up’, reflecting their expression status in *henn-1(pk2295)* mutant animals relative to wild-type animals. Statistical significance between N2 and *henn-1* mutant datasets was assessed with a Wilcoxon rank sum test (paired) and found to be highly significant (p<10^-16^). Similarly, non-paired Wilcoxon rank sum testing of the 22G coverage between ‘Down’ and ‘All’, and ‘Up’ and ‘All’ also revealed highly significant differences (p<10^−16^).

Finally, we performed micro array analysis on young adult animals to see how *henn-1* affects gene expression and how this relates to the effects we see on sRNA populations. Two technical replicates on two biological samples were analyzed and we considered genes to be significantly affected when the p-values in both arrays were below 0.05 and the change in expression level was at least two-fold. This resulted in a set of 160 genes that are up- and 267 genes that are down-regulated (Table S3) in *henn-1(pk2295)* animals. We analyzed the nature of these genes by asking for enrichment of gene expression domains as defined by Kim *et al.* [47]. Up-regulated genes are enriched for functions related to collagen while down-regulated genes are enriched for germline and embryonic expression, including 16 out of the 104 top-CSR-1 targets (Figure 6A, Table S3). Interestingly, when we relate the array data to our sRNA sequencing data (the TAP treated wild-type and *henn-1(pk2295)* libraries), we find that down-regulated genes are relatively rich in 22G RNA coverage, while up-regulated genes are relatively 22G RNA deprived (Figure 6B). In both up- and down-regulated gene classes the 22G RNA coverage drops approximately 30% upon loss of HENN-1. These data suggest that the observed expression changes are not triggered through altered 22G RNA levels, but are more likely an indirect effect of loss of HENN-1.

Next, we specifically analyzed ALG-3/4 and ERGO-1 target genes. The vast majority of the 399 tested ALG-3/4 targets did not change expression level. None were up-regulated and only three were found to be down-regulated (Table S3). The read counts for 26G RNAs mapping to these three genes were too low to derive meaningful correlations, and 22G counts remained either the same or dropped slightly. We therefore conclude that loss of HENN-1 does not directly affect ALG-3/4 target gene expression levels in young adult hermaphrodites. It should be noted, however, that ALG-3/4 targets are generally expressed during spermatogenesis, a stage that we have not specifically analyzed.

Out of 57 ERGO-1 targets tested, we only detect one with significantly changed expression levels. This gene, Y17D7B.4, is up-regulated in *henn-1(pk2295)* animals, accompanied by a 4-fold decrease in 26G RNA coverage and a two-fold drop in 22G RNA (Table S3). In fact, Y17D7B.4 is the only gene among the up-regulated genes with significant 26G and 22G coverage. Although ERGO-1 target genes do not display general up-regulation in *henn-1* mutant animals, it is striking that ERGO-1 targets do not follow the general down-regulation of germline-expressed genes, such as for example CSR-1 target genes.

## Discussion

We have addressed the *C. elegans* HEN1 ortholog HENN-1 in terms of its activity and its sub-cellular localization and have described its effect on the various endogenous RNAi pathways of this nematode. The major impact of HENN-1 is on the ERGO-1 26G RNAi pathway, while ALG-3/4 26G RNAs are unaffected. Strikingly, 21U RNAs are major targets of HENN-1, yet their stability in the germline is only marginally affected by loss of HENN-1. Below, we further discuss the implications of these findings.

### Subcellular localization of HENN-1

Using a GFP-tagged HENN-1 protein we have been able to demonstrate the presence of HENN-1 in granules in the germline. These granules overlap partly with PGL-1 foci, suggesting that HENN-1 is in, or at least near P-granules. During oogenesis HENN-1 is more diffuse and is also observed within the nucleus. This is a pattern we also observe in zebrafish, suggesting that this differential localization in oocytes may be functionally relevant. Since HENN-1 functions during the biogenesis of 21U and 26G RNAs this suggests that both these small RNA types are made within P-granules, the nematode version of an electron-dense and mitochondria-rich material known as nuage or germ-plasm in other organisms. This is consistent with many previously published findings regarding sRNA biogenesis in diverse model organisms, including *C. elegans* (reviewed in [5]).

### HENN-1 and exogenous RNAi

We describe an intriguing effect of HENN-1 on RNAi triggered through exogenous dsRNA triggers. While loss of HENN-1 diminishes the efficiency of RNAi in the germline, it induces an RNAi hypersensitivity defect in the soma. The RNAi hypersensitivity in *henn-1* mutants may be readily explained by the fact that mutations in the ERGO-1 pathway generally result in this phenotype [23], [24], [42]–[45]. It has been proposed that hypersensitivity arises by reduced competition for shared components between the endoRNAi pathway mediated by ERGO-1 and the exoRNAi pathway mediated by the Ago protein RDE-1. However, why would *henn-1* be required for exoRNAi in the germline? Perhaps this germline RNAi resistance is related to our observation that many germline genes are expressed at lower levels. This may include lower expression of exoRNAi related factors in *henn-1* mutant animals. Indeed, seven different genes (*mex-1*, *mex-3*, *oma-1*, *puf-5*, *pie-1, egg-3* and *pos-1*) whose protein products are known to reside in P-granules are reduced. This may lead to reduced P-granule functionality and consequently reduced RNAi. Even more strikingly, two genes previously implicated directly in germline exoRNAi and co-suppression, *ppw-2 (wago-3)* [48] and *mut-7* [49], are approximately three-fold down in *henn-1* mutants (Table S3), providing a potential explanation for the opposing effects of *henn-1* on exoRNAi in the germline and the soma.

### Function for HENN-1 in 26G pathways

We have shown that ERGO-1-bound 26G RNAs are methylated by HENN-1. Methylation of these 26G RNAs has a stabilizing effect and prevents non-templated uridylation. In other systems, HEN1 homologs have similarly been implicated in sRNA stabilization, mostly in the context of target RNA recognition [17], [19]. Since 26G RNAs have targets that display perfect complementarity, this result is consistent with these previous findings.

We have also shown that in contrast to ERGO-1-bound 26G RNAs, ALG-3/4-bound 26G RNAs are not methylated. What could be the cause behind this difference? First, the cell-type producing 26G RNAs may have an effect on their methylation status. ALG-3/4 bound 26G RNAs are present during spermatogenesis, while ERGO-1 26G RNAs are present in the female germline and the embryo. This would imply that HENN-1 activity during spermatogenesis is low or absent. Second, 26G RNAs may become methylated outside the context of Ago proteins, after which ERGO-1 would preferentially bind methylated and ALG-3/4 non-methylated versions. Indeed, PAZ domains of different Ago proteins that bind methylated or non-methylated sRNAs have differential affinities for methylated or non-methylated 3′ ends of sRNAs [20]. While these two options are certainly possible, we favor a third explanation for the difference between ERGO-1 and ALG-3/4 26G RNA behavior. Interestingly, ERGO-1 is related in sequence to the Piwi proteins PRG-1 and PRG-2 that both bind methylated 21U RNAs, while ALG-3 and ALG-4 are more related to the Ago proteins ALG-1 and ALG-2 that are known to bind non-methylated miRNAs [34]. Therefore, it is likely that the type of Ago protein binding the 26G RNA is instructive in determining the 3′ end methylation status. Similarly, in *Drosophila*, one and the same sRNA sequence can be either methylated or not, based on the Ago protein by which it is bound [13], [14], [19], [50]. It is presently unclear why ERGO-1-type 26G RNAs would require HENN-1-mediated methylation and stabilization while ALG-3/4-type 26G RNAs do not.

Loss of HENN-1 results in rather modest effects on 26G dependent 22G RNAs and gene expression levels. While direct targets of the ERGO-1 pathway do display a drop in 22G RNAs, this could not be distinguished from a more global effect that decreases the 22G RNA coverage of many other germline expressed genes. This overall drop in 22G RNAs derived from germline expressed genes is accompanied by a general decrease in mRNA from these same genes. Given these global effects it is difficult to dissect direct effects of HENN-1 on published ERGO-1 target genes. However, as ERGO-1 target genes escape the overall trend of decreased expression of germline-expressed genes, loss of HENN-1 may indeed trigger direct up-regulation of ERGO-1 targets through a loss of ERGO-1 triggered 22G RNAs.

### Function for HENN-1 in the 21U RNA pathway

Finally, we describe a number of findings regarding the function of HENN-1 in the *C. elegans* Piwi pathway, or PRG-1 pathway. First, we do not find indications for stable interaction between HENN-1 and PRG-1. We have been unable to co-IP both proteins and using gel-filtration experiments we observe HENN-1 and PRG-1 in distinct complexes. Regarding the first observation, we have similarly been unable to co-IP Hen1 and Zili from zebrafish gonadal extracts (unpublished data). This makes us believe that the previously published interactions between Hen1 and specific Ago proteins in *Drosophila*, which were based on GST-pull-down experiments [14], reflect transient interactions rather than stable complexes. Interestingly, we show that HENN-1 is found in a complex of approximately 100 kDa, reflecting oligomerization and/or stable association with another, as yet unknown factor. Such factors could be other Ago proteins such as ERGO-1. However, we favor the interpretation that HENN-1 binds a non-Ago protein. Possibly, this could be a nuclease involved in trimming the 3′ ends of 21U RNAs. In this scenario, HENN-1 activity may be directly coupled to 3′ end formation during 21U RNA biogenesis, as has been suggested for piRNA biogenesis in silk-moth derived cell-free extracts [18].

Surprisingly, *henn-1* mutant animals display only minor effects on the global stability of 21U RNAs in the germline. This conclusion is based on 21U RNA abundance on Northern blots and on cloning frequencies analyzed through deep-sequencing. sRNA stability has been coupled to target recognition through a mechanism that likely involves the extraction of the 3′ end of sRNAs from the PAZ domain upon target RNA binding [19]. Typically, these interactions involve extensive base-pairing between sRNA and target RNA molecules. However, also when presented with a perfectly matching transgenic target RNA, the 21U-RNA species 21UR1 is not significantly destabilized, indicating that target dependent effects on the stability of 21U RNAs are limited. We note that *henn-1* does affect 21U abundance in the embryo [51], [52], suggesting that 2′O-methylation plays a role in the stabilizing 21U RNAs over longer time intervals. The only significant effect of loss of *henn-1* on 21U RNAs in the germline is on their length: significantly more 20-mer species are formed in absence of HENN-1. Therefore, the major function of 2′-O-methylation of 21U RNAs is to stabilize their extremely strict length of 21 nucleotides and not to prevent target dependent 21U-uridylation and de-stabilization. Since we show that *henn-1* mutant animals display defects in 21UR1-mediated silencing while 21UR1 levels remain unchanged, the maintenance of 21U RNAs as methylated 21-mers may be important for full PRG-1 activity. However, it should be kept in mind that 21UR1 sensor silencing depends on a downstream pathway mediated by factors that are shared with exoRNAi pathways, and we show that exoRNAi efficiencies are reduced in *henn-1* mutant animals. This provides an alternative, and less direct explanation for the reduced silencing of the 21UR1 sensor in *henn-1* mutant backgrounds.

The 21U pathway in *C. elegans* is poorly characterized in terms of endogenous targets. Almost no targets displaying extensive complementarity to 21U RNAs are present [29], [30], a feature that is different from piRNA systems in other organisms. Yet, *prg-1* mutant animals display clear phenotypes [29]–[31] suggesting that either PRG-1 has 21U-independent functions or that PRG-1:21U complexes regulate target RNAs through non-perfect base-pairing interactions. Interestingly, in a recent paper precisely such mismatched 21U RNA-target RNA interactions have been described [32]. Perhaps it is this lack of extensive target complementarity that has allowed the PRG-1 pathway to become uncoupled from target dependent 21U RNA modifying activities. Everything considered, it remains to be fully elucidated why *C. elegans* 21U RNAs are methylated.

## Methods

### Worm strains

Bristol N2 was the wild type strain used in this study. We used two *henn-1* alleles, *pk2295* and *pk2452*, a premature stop (R347X) and a missense mutant (D151N) respectively. Strains carrying these alleles have been described before [39]. Other alleles used in this study: *prg-1(pk2298), glp-4(bn2), mut-7(pk204)* and *mut-7(xf0007)* (a new *mut-7* allele, Q131X, picked up in a 21UR1 sensor activation screen in a *henn-1(pk2295)* mutant background). The 21UR1 sensor transgene is described by Bagijn *et al.* [32]. A strain expressing HENN-1::GFP was made by MosSCI based transgenesis [53] using pCFJ150, modified to include the *pgl-3* promoter, a C-terminal fusion of GFP to HENN-1 and the *tbb-2* 3′UTR [54].

### *In vitro* methyltransferase activity assay

GST, GST-HENN-1 and GST-HENN-1(D151N) were expressed in *E. coli* and purified over GST-beads as described before [17]. The missense mutation was introduced by side directed mutagenesis of the vector (Stratagene). Equal amounts of protein were loaded on a PAGE-SDS gel and stained with PageBlue according to Manufacturer directions. The methyltransferase assay was performed as described previously (Yu et al, 2005). A 100 µl reaction containing 50 mM Tris–HCl (pH 8.0), 100 mM KCl, 5 mM MgCl2, 0.1 mM EDTA, 2 mM DTT, 5% glycerol, 80U RNasin, 0.5 mCi S-adenosyl-L-[methyl-14C] methionine (Amersham), 5 µg purified protein (GST or GST-HENN-1), and 1 nmol of RNA substrate was incubated for 2 h at 37°C. The reaction was stopped with proteinase K for 15 min at 65°C after which it was extracted by phenol/chloroform. Small RNAs were precipitated with ethanol and analyzed on a 12% acrylamide gel. The gel was treated with an autoradiography enhancer (En3hance, Perkin Elmer) and exposed to X-ray film at −80°C. Sequences of synthetic piRNAs were as described before [17]. Sequences of RNA oligos used with different 3′ end nucleotides: 5′- UGAGGUAGUAGGUUGUAUAGU-U/A/G/C.

### Antibodies

Polyclonal HENN-1 antibodies were raised in rabbits, following injection of synthetic peptides (Eurogentec, DoubleXP protocol). The peptide sequence yielding functional HENN-1 antibodies was: MAHTSDGWGAPYDNQ-C (cysteine was added for coupling to KLH carrier). We generated rabbit polyclonal antibodies against PRG-1 using Peptide Specialty Laboratories (PSL, Heidelberg, Germany) following the 2× Epitope Immunisation protocol. Peptides used for immunization are: Antigen 1: SGRGRGRGSGSNNSGGKDQKYL-C, Antigen 2: RQQGQSKTGSSGQPQKC. We purified sera using an antigen 2-coupled peptide column and used purified IgGs at 1∶4,000 dilution in Western blot. PGL-1 antibody was obtained from DSHB (OIC1D4).

### Northern blot and B-elimination

Total RNA was isolated using RNA lysis buffer and Trizol. Subsequently, small RNA was isolated with the Mirvana kit. Northern blotting was done as described previously [17]. 20 µg of small RNA was loaded on a 12% polyacrylamide gel and blotted according to standard procedures. Probe sequences: 21U-R1: GCACGGTTAACGTACGTACCA siR26-263: TAGCATATGCATGCACCATAAACAAC *let-7*: AACTATACAACCTACTACCTCA. 22G-1: AAAGTGGTCAAGCACGGTTAAC 22G-2: AGTAAACCCAGCTTTCTTGTAC.

Ambion hybridization buffer (ULTRAhyb-Oligo) was used. To test the presence of the 3′-end modification of 21U-RNAs and 26G RNAs, 20 µg small RNA was treated with NaIO4 followed by β-elimination as described previously [41]. Blots were exposed to phosphor-imager screens that were scanned on a BAS-2500 imager. Blots were analyzed using ImageQuant software.

### Immuno staining

Immuno staining protocols were followed as described before [22].

### RNAi

RNAi was performed as described [55], using bacterial strains expressing dsRNA for the indicated target genes.

### Library preparation

RNA was isolated following fast proteinase K mediated lysis of the animals followed by adding 3 volumes of Trizol LS. Further RNA isolation was as prescribed by the manufacturers (Invitrogen). Oxidation of the RNA was done as described before [41]. Small RNA libraries were prepared as described before [17], using 4-base bar-codes (ACTA or TACA) to allow sequencing of two samples in one lane of an Illumina GAII sequencer.

### Sequence analysis

Raw sequence data were preprocessed to split reads according to barcodes and trim 3′ adapters. After grouping identical reads to remove redundancy, processed reads were mapped to the WS220 *C.elegans* genome assembly using MEGABLAST software [56] requiring perfect matching of at least the 18 first nucleotides. Non-matching 3′ nucleotides were trimmed and recorded for the calculation of non-templated read modification. Genomic annotations of mapped loci, including 21U RNAs, were retrieved from Ensembl database (v.62) using custom scripts and Ensembl API [57].

### Microarrays

Custom 1×22 k arrays for *C. elegans* from Agilent were used according to manufacture′s protocol. 0.5 µg of total RNA from young adults was converted into cRNA and labeled with Cy3 or Cy5. Samples were subsequently hybridized overnight and washed. A dye swap was included as a technical replicate. The whole experiment was done in duplicate. The data was analyzed using Array Assist and Feature extract software from Agilent. Expression domain analysis was performed using the following web-site:http://nemates.org/gl/cgi-bin/gl_mod.cgi?action=compare2

### Data submission

Illumina sequencing data has been deposited at GEO, submission number GSE31783.

## Supporting Information

Figure S1 Protein sequence alignment of HENN-1 and homologs. Protein sequence alignment of *C. elegans* HENN-1 (C02F5.6, splice variants a and b). The catalytic domain is underlined and conserved residues are labeled with an * (identical), : (very similar) or . (similar). The two mutated residues are indicated in the figure.
(PDF)

Figure S2 Brood size analysis of *henn-1* mutants. Brood size and survival analysis of wild-type and *henn-1* mutant strains.
(PDF)

Figure S3 Effect of *henn-1* on 26G RNA. Northern blot for siR26–263 as shown in Figure 1, but enhanced using Photoshop.
(PDF)

Figure S4 Effect of *henn-1* on exoRNAi. (A) RNAi against *sqt-3* in wild-type (WT), *henn-1(pk2295)* and *henn-1(pk2295;henn-1::GFP* animals, scoring for a roller phenotype. The effect of loss of *sqt-3* can be variable, depending on temperature and age of the animals and genetic alleles of *sqt-3* display complex behavior [58], [59]. For example, *sqt-3(sc63)* heterozygous animals are rollers, while *sqt-3(sc63)* homozygotes are normal moving [58]. Therefore the apparent resistance of *henn-1* mutant animals to *sqt-3* may in fact reflect an Eri phenotype. In fact, in one sub-optimal RNAi experiment we obtained rollers with henn-1 mutant animals, while WT animals displayed no phenotype. This suggests that the differences observed in the data presented are likely caused by RNAi hypersensitivity, rather than RNAi resistance. The differences between *henn-1* and WT and *henn-1 and henn-1; henn-1::GFP* are statistically significant (p<0.005, T-test, n = 4). (B) RNAi against *lir-1* in wild-type (WT), *henn-1(pk2295)* and *henn-1(pk2295;henn-1::GFP* animals scoring for survival until the L1 stage. The differences between *henn-1* and WT and *henn-1 and henn-1; henn-1::GFP* are statistically significant (p<0.0005, T-test, n = 5). (C) RNAi against *pop-1* in wild-type (WT), *henn-1(pk2295)* and *henn-1(pk2295;henn-1::GFP* animals, scoring for burst and protruding vulva phenotypes. The difference between *henn-1* and WT is statistically significant (p<0.0005, T-test, n = 5). The vulva phenotype is not significantly rescued by the *henn-1::GFP* transgene, likely because the *pgl-3* driven expression of *henn-1::GFP* does not reach into the vulva lineage. (D) RNAi against *pop-1* in wild-type (WT), *henn-1(pk2295)* and *henn-1(pk2295;henn-1::GFP* animals. Although the vulva phenotype is not rescued, the complete sterility triggered by *pop-1* in *henn-1* animals is rescued by the *pgl-3:henn-1::GFP* transgene is visualized by the appearance of embryos on the plate. Burst and protruding vulvae are indicated by red and black asterisks respectively. (E) RNAi against *gpb-1* in wild-type (WT), *henn-1(pk2295)* and *henn-1(pk2295;henn-1::GFP* animals, scoring for embryonic survival until L1 stage. The differences between *henn-1* and WT and *henn-1 and henn-1; henn-1::GFP* are statistically significant (p<0.05 and p<0.005 respectively, T-test, n = 5).
(PDF)

Figure S5 Western blot analysis of HENN-1::GFP transgenic animals. Western blot analysis of the indicated *C. elegans* lines with an anti-GFP antibody. ‘Free GFP’ indicates a protein that may represent GFP that has become separated from the HENN-1::GFP fusion protein.
(PDF)

Figure S6 HENN-1 and PRG-1 expression. (A) Images of GFP::PRG-1 germline nuclei in wild-type and *henn-1(pk2295)* animals. (B) Western blot for HENN-1, PRG-1 and tubulin on samples derived from wild-type and *henn-1(pk2295)* animals.
(PDF)

Figure S7 Northern blot for Y51G11C.51. Northern blot probed for Y51G11C.51 small anti-sense RNAs, using a mixture of DNA oligo nucleotides covering Y51G11.51. Signals are weak, but 26G and 22G RNA signals can be detected. In the *henn-1* mutant samples the 26G signal is non-detectable anymore, while 22G RNA signal is still present, although weaker.
(PDF)

Figure S8 Non-templated bases on 22G RNAs. Bar diagram displaying the frequencies of non-templated base additions found on 22G reads, as a percentage of the total 22G read count.
(PDF)

Table S1 Read counts obtained for the various sequenced libraries. The numbers represent the numbers of reads obtained within each category. siRNA' contains all reads complementary to annotated mRNAs. ‘senseRNA’ contains reads from mRNAs of sense polarity. The siRNA category holds both the 22G and 26G RNAs further discussed in this work. These two classes are not individually represented in this table. ‘Other’ category contains reads that partly overlap annotated transcripts and reads that overlap non-annotated transcripts, including potential non-annotated miRNAs. WT: wild-type. ox: RNA was oxidized with NaIO4 before cloning (enriches for 2′O-methylated small RNAs). Note that the mapped reads from these libraries are much lower than non-oxidized libraries. This is caused by a high fraction of adaptor-only reads in the oxidized libraries, presumably caused by the fact that most other RNAs have become unclonable. tap: treated with TAP enzyme before cloning (removes 5′-tri-phosphates).
(PDF)

Table S2 21U, 22G, and 26G species counts. This table displays the number of species sequenced for the three small RNA classes listed, irrespective of how often each species has been sequenced. The ‘Total mapped reads’ column reflects the total number of raw reads for these three small RNA species (also see Table S1).
(PDF)

Table S3 Normalized read counts in reads per million (rpm). The classes in this table represent reads not mapping to structural RNAs such as tRNAs or rRNAs. These together have been set to 1 million. The conversion factors for each library are given in the last column.
(PDF)

Table S4 This table lists annotated genes from the *C.elegans* genome and gives displays the expression ratios from the two micor array experiments described in the paper. In addition it lists the normalised read counts of small RNAs found in the diverse libraries. WT: wild-type, ox: oxidized(enriched for 2′O-methylated RNAs), tap: tap treated RNA (allows cloning of 5′-triphosphate RNAs). Status indicates to which specific Argonaute small RNAs have been found to bind, based on published literature (see main text).
(XLSX)
