# Supplementary material for: Differential Impact of the HEN1 Homolog HENN-1 on 21U and 26G RNAs in the Germline of Caenorhabditis elegans
Source: PLoS Genet. 2012 Jul 19;8(7):e1002702. doi: 10.1371/journal.pgen.1002702 (PMC3400576; doi:10.1371/journal.pgen.1002702)
Supplement: Figure S1 — Protein sequence alignment of HENN-1 and homologs. Protein sequence alignment of C. elegans HENN-1 (C02F5.6, splice variants a and b). The catalytic domain is underlined and conserved residues are labeled with an * (identical), : (very similar) or . (similar). The two mutated residues are indicated in the figure. (PDF) [file pgen.1002702.s001.pdf]

```
C02F56a       MAHTSDGWGAPYDNQTYVEAYEQLEIALLEPLDRILETANVEEFRPKFKNHQDPNDRKNK 60
C02F56b       MAHTSDGWGAPYDNQTYVEAYEQLEIALLEPLDRILETANVEEFRPKFKNHQDPNDRKNK 60
dmhen1        ------------------------------------------------------------
henmt1        ------------------------------------------------------------

C02F56a       KNNDEEWRDSIYNIATDESDTDDHEQRKNFFQPPLQVQRNSFVKNTLMEFKRSSQIDISR 120
C02F56b       KNNDEEWRDSIYNIATDESDTDDHEQRKNFFQPPLQVQRNSFVKNTLMEFKRSSQIDISR 120
dmhen1        ----------MFSHKFICGSLTKMTETGITFDPPVYEQRYCATIQILEDARWKD--QIKK 48
henmt1        -------------------------MTATPFSPPLYMQRYQFVIDYVKTYRPRK------ 29
                                            *.**:  **   . : :   :  .

C02F56a       LAVMGCGEMSLEKGICEYLGSFGTINVLSVDIDEPSLSIGQQLLGKAESIFLRKHLERNA 180
C02F56b       LAVMGCGEMSLEKGICEYLGSFGTINVLSVDIDEPSLSIGQQLLGKAESIFLRKHLERNA 180
dmhen1        VVEFGCAEMRFFQ-LMRRIETIEHIG--LVDIDKSLLMRNLTSVNPLVSDYIRSRAS--- 102
henmt1        VIDFGCAECCLLKKLKFHRNGIQLLVG--VDINSVVLLKRMHSLAPLVSDYLQPSDG--- 84
              :  :**.*  : : :      :  :    ***:.  *      :    * :::

C02F56a       EILAVETGLPVLMRSYVGDILEPDHRFADVDAIVSMEVVEHIPLPNAKKFVENVLGTLMP 240
C02F56b       EILAVETGLPVLMRSYVGDILEPDHRFADVDAIVSMEVVEHIPLPNAKKFVENVLGTLMP 240
dmhen1        ---------PLKVQILQGNVADSSEELRDTDAVIAIELIEHVYDDVLAKIPVNIFGFMQP 153
henmt1        ---------PLTIELYQGSVMEREPCTKGFDLVTCVELIEHLELEEVERFSEVVFGYMAP 135
                       *: :.   *.: : .    . * : .:*::**:      ::   ::* : *

C02F56a       RIFIFSTPNHEYNAVFG----MEPGEFRHGDHKFEMNRKEFSNWLEELSIRFPHYQIDPP 296
C02F56b       RIFIFSTPNHEYNAVFG----MEPGEFRHGDHKFEMNRKEFSNWLEELSIRFPHYQIDPP 296
dmhen1        KLVVFSTPNSDFNVIFTRFNPLLPNGFRHEDHKFEWSRDEFKNWCLGIVEKYPNYMFSLT 213
henmt1        GAVIVTTPNAEFNPLLPG-----LRGFRNYDHKFEWTRAEFQTWAHRVCREHG-YSVQFT 189
                .:.:*** ::* ::          **: ***** .* **..*   :  ..  * .. .

C02F56a       HYIGMTRGYENLSGASQAAVCR-----LQVDLN------TTLPQEVTPYEMVG--HLPCR 343
C02F56b       HYIGMTRGYENLSGASQAAVCR-----LQVDLN------TTLPQEVTPYEMVG--HLPCR 343
dmhen1        GVGNPPKEYESVGPVSQIAIFVR-KDMLEMQLVNPLVSKPNIDKESIPYKLIHTVEYPFY 272
henmt1        GVGEAAGHWRDVGFCTQIAVFQRNFDGVNRSMS------NAEHLEPSVYRLLYRVVYPSL 243
                   .  :..:.  :* *:       :: .:            *   *.::     *

C02F56a       LGSRLIAYNLVKEAFLDWLEKIELQEHEP----------------RTDG-YSPYWIFNVQ 386
C02F56b       LGSRLIAYNLVKEAFLDWLEKIELQEHEP----------------RTDG-YSPYWIFNVQ 386
dmhen1        VDTRTEKEKLWTEVQIELQRFKRQFESSE----------------IEEGTYQDTCNMPIA 316
henmt1        CDNNIYQKTLINEVLYEAQHLRQQWLIRENMNNNAHFYSPPLMEALHHGAEGNACEQQPV 303
               ...    .* .*.  :  .  .                        .*

C02F56a       NILHHLKAP-----------VSFALTIDEKVAMQVSS----------------------- 412
C02F56b       NILHHLKAP-----------VSFALTIDEKVAIKYIQGMTSRKVHAEYSHGFNGIVILQM 435
dmhen1        FLLDRLEHVGATKERIEELLLENNLTVENECVLIVSSDQESEWSDPYKFSDRSSQDDALV 376
henmt1        YQQGGIICVPLARVWSCPRVQALCGSLQRLREKLLEDERVRMSADGSALNLPADDDDDNV 363
                   :                   :::.       .

C02F56a       -----VIGTVEF---------------------------- 419
C02F56b       HSKEELIKTVQDNTL------------------------- 450
dmhen1        DQEQEEERWDQGPES------------------------- 391
henmt1        EEEEEEEEEENQQNVKAVSGAVNNMEEDWDRELGSYGDE 402
                        :
```

Methyltranserase domain based on Tkaczuk, BMC 2006

henn-1 pk2452

henn-1 pk2295

Mg2+ sites

Kamminga_Figure S1
