# Supplementary figures and images for: Differential Impact of the HEN1 Homolog HENN-1 on 21U and 26G RNAs in the Germline of Caenorhabditis elegans
Source: PLoS Genet. 2012 Jul 19;8(7):e1002702. doi: 10.1371/journal.pgen.1002702 (PMC3400576; doi:10.1371/journal.pgen.1002702)

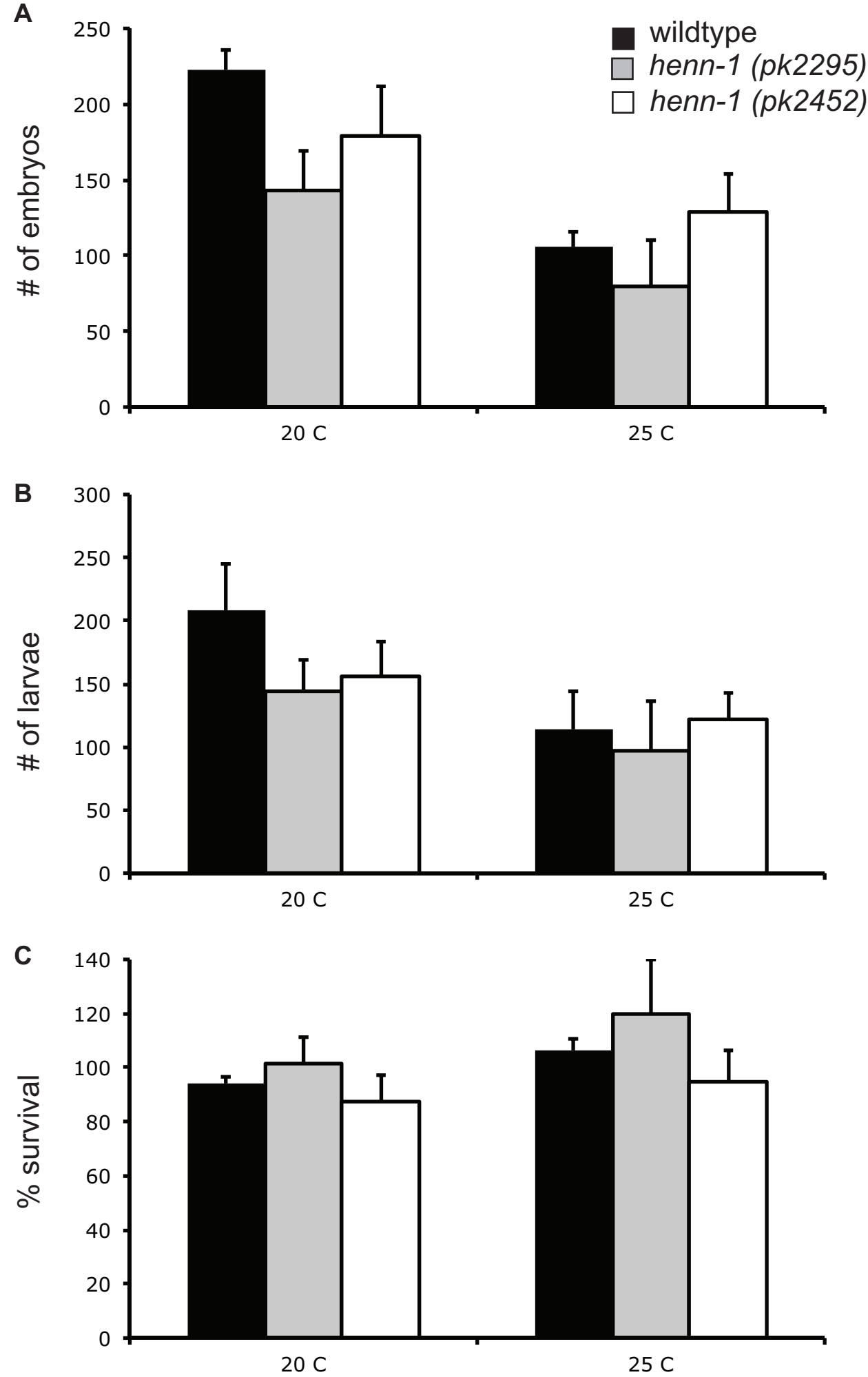

Supplement: Figure S2 — Brood size analysis of henn-1 mutants. Brood size and survival analysis of wild-type and henn-1 mutant strains. (PDF) [file pgen.1002702.s002.pdf]

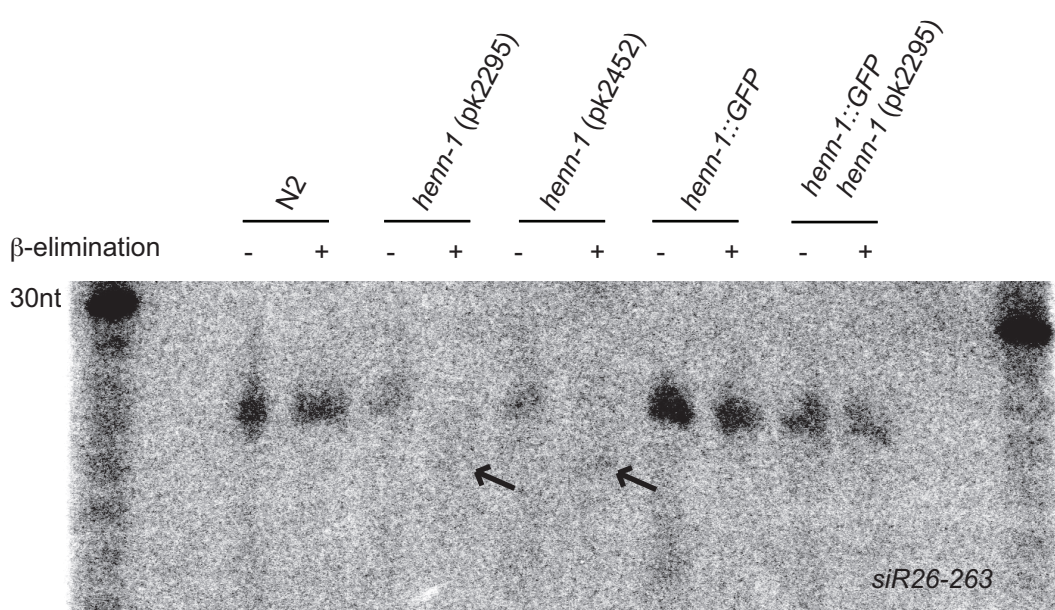

Supplement: Figure S3 — Effect of henn-1 on 26G RNA. Northern blot for siR26–263 as shown in Figure 1, but enhanced using Photoshop. (PDF) [file pgen.1002702.s003.pdf]

**A**

*sqt-3* RNAi

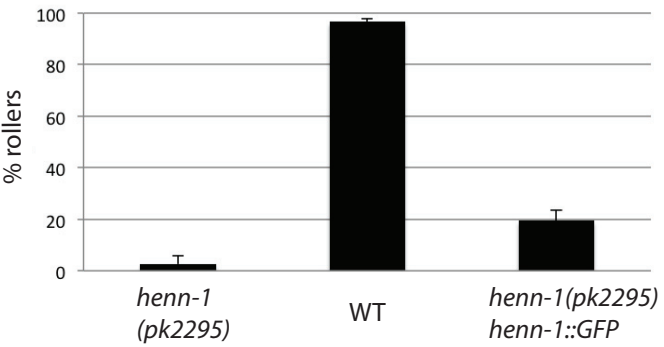

**B**

*lir-1* RNAi

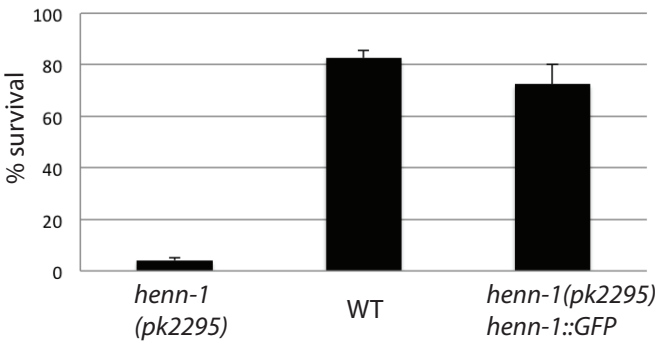

**C**

*pop-1* RNAi

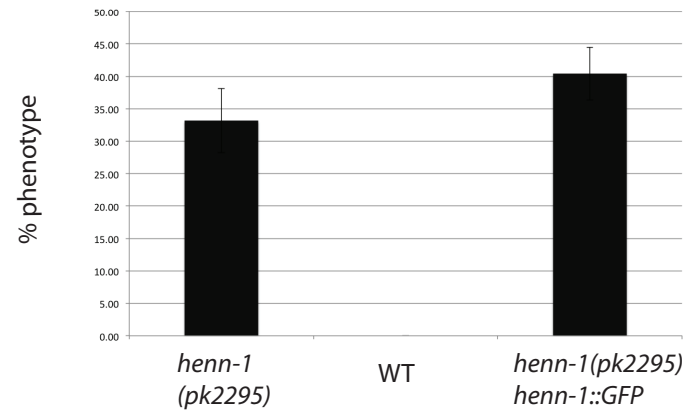

**E**

*gpb-1* RNAi

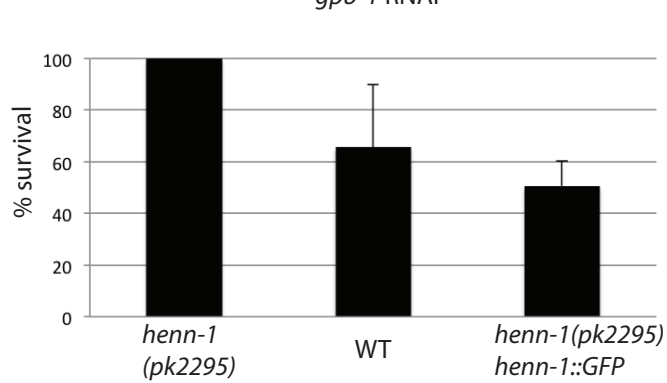

**D**

*henn-1(pk2295)*

WT

*henn-1(pk2295); henn-1::GFP*

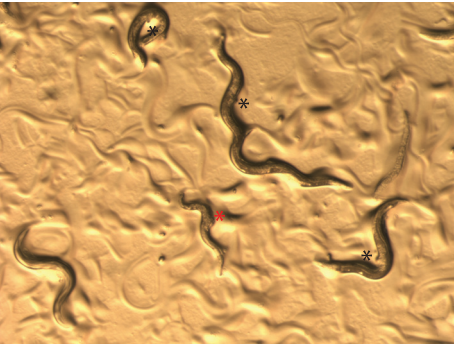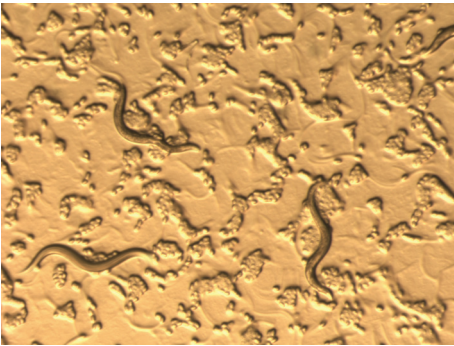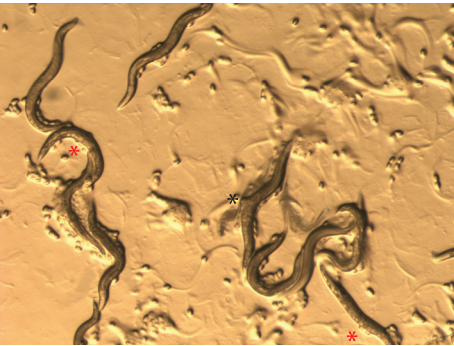

Supplement: Figure S4 — Effect of henn-1 on exoRNAi. (A) RNAi against sqt-3 in wild-type (WT), henn-1(pk2295) and henn-1(pk2295;henn-1::GFP animals, scoring for a roller phenotype. The effect of loss of sqt-3 can be variable, depending on temperature and age of the animals and genetic alleles of sqt-3 display complex behavior [58], [59]. For example, sqt-3(sc63) heterozygous animals are rollers, while sqt-3(sc63) homozygotes are normal moving [58]. Therefore the apparent resistance of henn-1 mutant animals to sqt-3 may in fact reflect an Eri phenotype. In fact, in one sub-optimal RNAi experiment we obtained rollers with henn-1 mutant animals, while WT animals displayed no phenotype. This suggests that the differences observed in the data presented are likely caused by RNAi hypersensitivity, rather than RNAi resistance. The differences between henn-1 and WT and henn-1 and henn-1; henn-1::GFP are statistically significant (p<0.005, T-test, n = 4). (B) RNAi against lir-1 in wild-type (WT), henn-1(pk2295) and henn-1(pk2295;henn-1::GFP animals scoring for survival until the L1 stage. The differences between henn-1 and WT and henn-1 and henn-1; henn-1::GFP are statistically significant (p<0.0005, T-test, n = 5). (C) RNAi against pop-1 in wild-type (WT), henn-1(pk2295) and henn-1(pk2295;henn-1::GFP animals, scoring for burst and protruding vulva phenotypes. The difference between henn-1 and WT is statistically significant (p<0.0005, T-test, n = 5). The vulva phenotype is not significantly rescued by the henn-1::GFP transgene, likely because the pgl-3 driven expression of henn-1::GFP does not reach into the vulva lineage. (D) RNAi against pop-1 in wild-type (WT), henn-1(pk2295) and henn-1(pk2295;henn-1::GFP animals. Although the vulva phenotype is not rescued, the complete sterility triggered by pop-1 in henn-1 animals is rescued by the pgl-3:henn-1::GFP transgene is visualized by the appearance of embryos on the plate. Burst and protruding vulvae are indicated by red and black asterisks respectiv [file pgen.1002702.s004.pdf]

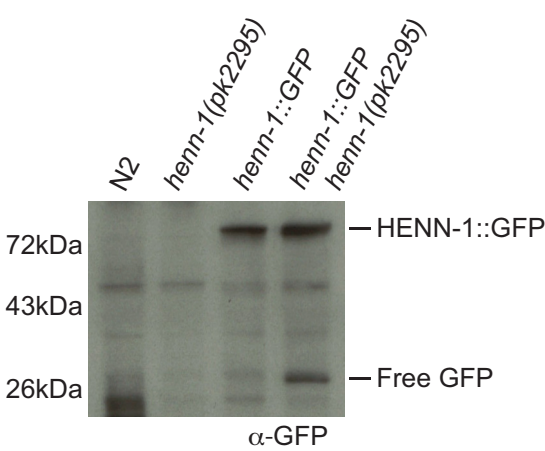

Supplement: Figure S5 — Western blot analysis of HENN-1::GFP transgenic animals. Western blot analysis of the indicated C. elegans lines with an anti-GFP antibody. ‘Free GFP’ indicates a protein that may represent GFP that has become separated from the HENN-1::GFP fusion protein. (PDF) [file pgen.1002702.s005.pdf]

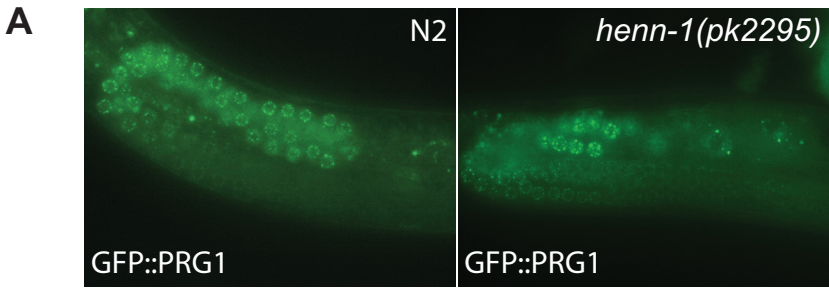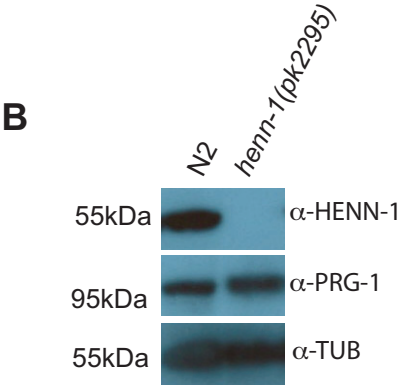

Supplement: Figure S6 — HENN-1 and PRG-1 expression. (A) Images of GFP::PRG-1 germline nuclei in wild-type and henn-1(pk2295) animals. (B) Western blot for HENN-1, PRG-1 and tubulin on samples derived from wild-type and henn-1(pk2295) animals. (PDF) [file pgen.1002702.s006.pdf]

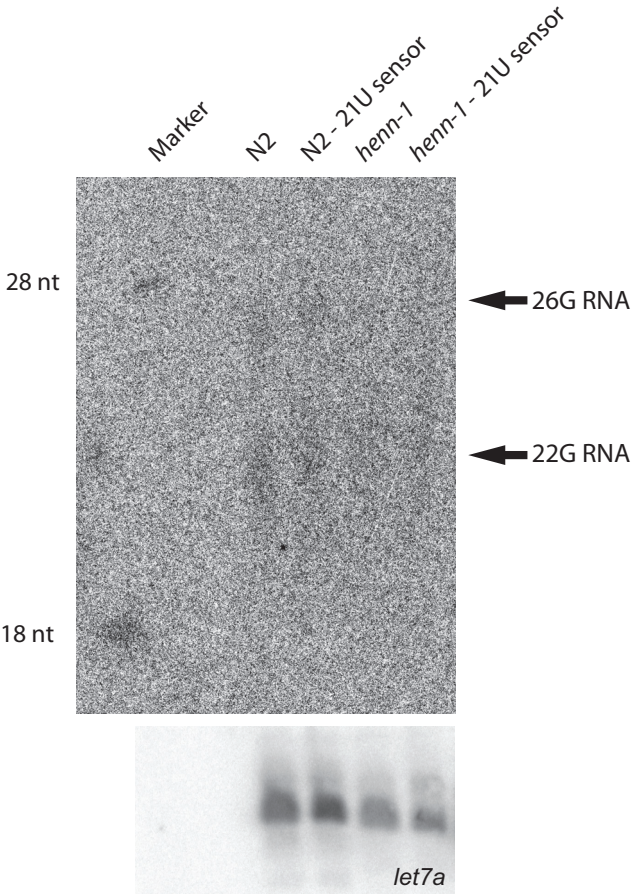

Supplement: Figure S7 — Northern blot for Y51G11C.51. Northern blot probed for Y51G11C.51 small anti-sense RNAs, using a mixture of DNA oligo nucleotides covering Y51G11.51. Signals are weak, but 26G and 22G RNA signals can be detected. In the henn-1 mutant samples the 26G signal is non-detectable anymore, while 22G RNA signal is still present, although weaker. (PDF) [file pgen.1002702.s007.pdf]

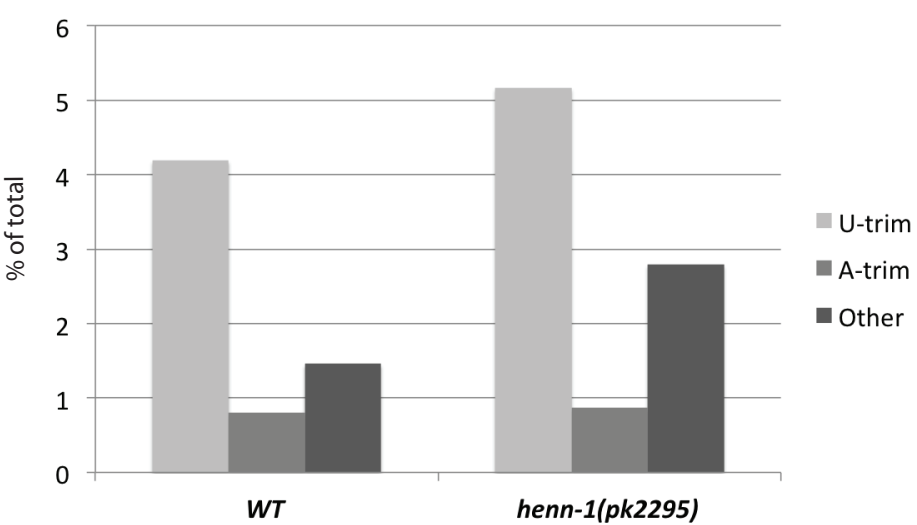

Supplement: Figure S8 — Non-templated bases on 22G RNAs. Bar diagram displaying the frequencies of non-templated base additions found on 22G reads, as a percentage of the total 22G read count. (PDF) [file pgen.1002702.s008.pdf]
