# Supplementary material for: Differential Impact of the HEN1 Homolog HENN-1 on 21U and 26G RNAs in the Germline of Caenorhabditis elegans
Source: PLoS Genet. 2012 Jul 19;8(7):e1002702. doi: 10.1371/journal.pgen.1002702 (PMC3400576; doi:10.1371/journal.pgen.1002702)
Supplement: Table S1 — Read counts obtained for the various sequenced libraries. The numbers represent the numbers of reads obtained within each category. siRNA' contains all reads complementary to annotated mRNAs. ‘senseRNA’ contains reads from mRNAs of sense polarity. The siRNA category holds both the 22G and 26G RNAs further discussed in this work. These two classes are not individually represented in this table. ‘Other’ category contains reads that partly overlap annotated transcripts and reads that overlap non-annotated transcripts, including potential non-annotated miRNAs. WT: wild-type. ox: RNA was oxidized with NaIO4 before cloning (enriches for 2′O-methylated small RNAs). Note that the mapped reads from these libraries are much lower than non-oxidized libraries. This is caused by a high fraction of adaptor-only reads in the oxidized libraries, presumably caused by the fact that most other RNAs have become unclonable. tap: treated with TAP enzyme before cloning (removes 5′-tri-phosphates). (PDF) [file pgen.1002702.s009.pdf]

	21U	22G RNA	26G RNA	miRNA	Repeat elements	Other	senseRNA	rRNA	snRNA	snoRNA	tRNA	Total mapped reads
**WT**	192,700	49,081	36,452	6,734,722	127,454	745,737	387,983	1,368,939	22,687	16,783	520,295	**10,202,833**
**WT ox**	146,952	1,992	37,467	77,221	10,454	288,784	10,340	13,806	9,595	495	10,865	**607,971**
**WT tap**	115,548	738,873	13,712	2,699,325	252,823	1,862,168	210,090	816,558	13,626	8,793	149,924	**6,881,440**
henn-1(pk2452)	126,953	23,429	12,393	5,220,280	89,114	469,834	461,637	1,090,623	13,951	13,143	238,672	**7760,029**
***henn-1(pk2452)* ox**	104,578	3,251	18,523	102,850	18,186	315,549	58,878	104,178	193,010	5,949	90,394	**1,015,346**
henn-1(pk2295)	95,509	17,713	3,763	3,900,782	66,721	277,961	264,862	775,284	10,503	8,580	245,226	**5,666,904**
***henn-1(pk2295)* ox**	1,354	163	45	30,368	2,491	33,009	14,531	27,723	48,490	1,142	22,798	**182,114**
***henn-1(pk2295)* tap**	86,131	494,688	2,881	3,402,017	181,503	1,352,107	327,595	1,330,082	16,147	10,492	175,856	**7,379,499**

Table S1. Read counts obtained for the various sequenced libraries.
The numbers represent the numbers of reads obtained within each category.
siRNA' contains all reads complementary to annotated mRNAs. 'senseRNA' contains reads from mRNAs of sense polarity.
The siRNA category holds both the 22G and 26G RNAs further discussed in this work. These two classes are not individually represented in this table.
'Other' category contains reads that partly overlap annotated transcripts and reads that overlap non-annotated transcripts, including potential non-annotated miRNAs.
WT: wild-type.
ox: RNA was oxidized with $NaIO_4$ before cloning (enriches for 2'O-methylated small RNAs).
Note that the mapped reads from these libraries are much lower than non-oxidized libraries.
This is caused by a high fraction of adaptor-only reads in the oxidized libraries, presumably caused by the fact that most other RNAs have become unclonable.

tap: treated with TAP enzyme before cloning (removes 5'-tri-phosphates).
