# Supplementary material for: Differential Impact of the HEN1 Homolog HENN-1 on 21U and 26G RNAs in the Germline of Caenorhabditis elegans
Source: PLoS Genet. 2012 Jul 19;8(7):e1002702. doi: 10.1371/journal.pgen.1002702 (PMC3400576; doi:10.1371/journal.pgen.1002702)
Supplement: Table S2 — 21U, 22G, and 26G species counts. This table displays the number of species sequenced for the three small RNA classes listed, irrespective of how often each species has been sequenced. The ‘Total mapped reads’ column reflects the total number of raw reads for these three small RNA species (also see Table S1). (PDF) [file pgen.1002702.s010.pdf]

	21U	22G RNA	26G RNA	Total mapped reads
**WT**	4,723	25,429	1,454	**278,233**
**WT ox**	3,807	1,304	910	**186,411**
**WT tap**	2,427	110,062	796	**868,133**
henn-1(pk2452)	5,228	14,840	898	**162,775**
***henn-1(pk2452)* ox**	5,308	1,060	669	**126,352**
henn-1(pk2295)	5,137	11,127	802	**116,985**
***henn-1(pk2295)* ox**	378	27	13	**1,562**
***henn-1(pk2295)* tap**	3,744	97,204	605	**583,700**

Table S2. 21U, 22G and 26G species counts.
This table displays the number of species sequenced for the three small RNA classes listed, irrespective of how often each species has been sequenced. The 'Total mapped reads' column reflects the total number of raw reads for these three small RNA species (also see Table S1).
