# Supplementary material for: Differential Impact of the HEN1 Homolog HENN-1 on 21U and 26G RNAs in the Germline of Caenorhabditis elegans
Source: PLoS Genet. 2012 Jul 19;8(7):e1002702. doi: 10.1371/journal.pgen.1002702 (PMC3400576; doi:10.1371/journal.pgen.1002702)
Supplement: Table S3 — Normalized read counts in reads per million (rpm). The classes in this table represent reads not mapping to structural RNAs such as tRNAs or rRNAs. These together have been set to 1 million. The conversion factors for each library are given in the last column. (PDF) [file pgen.1002702.s011.pdf]

	21U	22G	26G	miRNA	Repeat elements	Other	Reads-rpm conversion
**WT**	24,723	6,297	4,677	864,401	16,359	95,678	0.1283
**WT ox**	265,968	3,605	67,812	139,765	18,921	522,670	1.8099
**WT tap**	23,341	149,252	2,770	545,385	51,082	376,158	0.2020
henn-1(pk2452)	21,569	3,981	2,106	887,000	15,142	79,825	0.1699
***henn-1(pk2452)* ox**	193,940	6,029	34,351	190,732	33,725	585,186	1.8545
henn-1(pk2295)	22,091	4,097	870	902,258	15,433	64,292	0.2313
***henn-1(pk2295)* ox**	20,420	2,458	679	457,972	37,570	497,809	15.0810
***henn-1(pk2295)* tap**	17,252	99,086	577	681,427	36,355	270,827	0.2003

Table S2. Normalized read counts in reads per million (rpm).
The classes in this table represent reads not mapping to structural RNAs such as tRNAs or rRNAs. These together have been set to 1 million. The conversion factors for each library are given in the last column.
